# Understanding the Effect of Statins and Patient Adherence in Atherosclerosis via a Quantitative Systems Pharmacology Model Using a Novel, Hybrid, and Multi-Scale Approach

**DOI:** 10.3389/fphar.2017.00635

**Published:** 2017-09-13

**Authors:** Cesar Pichardo-Almarza, Vanessa Diaz-Zuccarini

**Affiliations:** ^1^Multiscale Cardiovascular Engineering Group (MUSE), Department of Mechanical Engineering, University College London London, United Kingdom; ^2^Institute of Healthcare Engineering, University College London London, United Kingdom

**Keywords:** atherosclerosis, statins, multiscale modeling, hybrid model, patient adherence, pharmacokinetics/pharmacodynamics (PKPD), quantitative systems pharmacology (QSP)

## Abstract

**Background and Objective:** Statins are one of the most prescribed drugs to treat atherosclerosis. They inhibit the hepatic HMG-CoA reductase, causing a reduction of circulating cholesterol and LDL levels. Statins have had undeniable success; however, the benefits of statin therapy crystallize only if patients adhere to the prescribed treatment, which is far away from reality since adherence decreases with time with around half of patients discontinue statin therapy within the first year. The objective of this work is to; firstly, demonstrate a formal *in-silico* methodology based on a hybrid, multiscale mathematical model used to study the effect of statin treatment on atherosclerosis under different patient scenarios, including cases where the influence of medication adherence is examined and secondly, to propose a flexible simulation framework that allows extensions or simplifications, allowing the possibility to design other complex simulation strategies, both interesting features for software development.

**Methods:** Different mathematical modeling paradigms are used to present the relevant dynamic behavior observed in biological/physiological data and clinical trials. A combination of continuous and discrete event models are coupled to simulate the pharmacokinetics (PK) of statins, their pharmacodynamic (PD) effect on lipoproteins levels (e.g., LDL) and relevant inflammatory pathways whilst simultaneously studying the dynamic effect of flow-related variables on atherosclerosis progression.

**Results:** Different scenarios were tested showing the impact of: (1) patient variability: a virtual population shows differences in plaque growth for different individuals could be as high as 100%; (2) statin effect on atherosclerosis: it is shown how a patient with a 1-year statin treatment will reduce his plaque growth by 2–3% in a 2-year period; (3) medical adherence: we show that a patient missing 10% of the total number of doses could increase the plaque growth by ~1% (after 2 years) compared to the same “regular” patient under a 1-year treatment with statins.

**Conclusions:** The results in this paper describe the effect of pharmacological intervention combined with biological/physiological or behavioral factors in atherosclerosis progression and treatment in specific patients. It also provides an exemplar of basic research that can be practically developed into an application software.

## Introduction

Atherosclerosis is a chronic disease that has become a burden in cardiovascular services being the leading cause of death in the western world. The clinical manifestation of atherosclerosis includes coronary artery disease (CAD), cerebrovascular disease (CVA), and peripheral arterial disease (PAD), having an incidence of 67% for men and 50% for women after the age of 40 (Robinson et al., 2009). The process of atherosclerotic plaque formation is very complex, multifactorial, and systemic. There is evidence from experimental findings supporting the fact that hemodynamic stimuli have an influence on physical properties of the arterial endothelium, e.g., permeability and cell geometry, likely causing an accumulation of lipoprotein macromolecules (e.g., Low Density Lipoproteins—LDL) and atherosclerosis progression. During the early stages of the disease, after monocytes infiltrate the arterial wall, lipid-laden macrophages (foam cells) accumulate in the subendothelium, following an accumulation of fibrous tissue and smooth muscles cells. The formation of the lesions is promoted by plasma proteins carrying elevated levels of cholesterol and triglycerides. On top of this, environmental factors are understood to play a role as well as genetic makeup (Fox et al., 2003). The framework that surrounds the atherosclerotic process is bewildering in complexity and extent. Understanding and managing these combined cause-effect relationships proves challenging for clinicians, who must deal with the disease and its consequences every day.

This is a case where integrative, multi-scale, mechanistic, computational models can offer much needed help. Disease simulation models can capture complex characteristics and mechanisms (albeit, simplified) in order to use an *in silico* approach for the analysis, exploration, and understanding of the development of these diseases (Alimohammadi et al., 2016, 2017; Donadoni et al., 2017). These mechanistic, simulation models offer strong interpretability and understanding of the underlying processes and their interplay in the development of the disease. They also offer the possibility to combine the disease model with more conventional approaches such as pharmacokinetics-pharmacodynamics (PK-PD) or physiologically-based pharmacokinetic models (PBPK) to understand two key aspects of medical treatment: (a) the effect of drugs on disease progression and (b) to explore diverse scenarios to analyze the effect of patient adherence.

The objective of this paper is to provide, for the first time, a detailed description of an integrative, multi-scale model (or Quantitative Systems Pharmacology—QSP model) used to (a) understand the effect of statins on the progression of atherosclerosis and (b) to understand the effect of patient adherence through multiple (possible) scenarios, all while describing in detail the formalisms and methodologies involved. Statins are amongst the most prescribed drugs worldwide and they are typically prescribed for a lifetime. This is a key issue as statins have well-known side-effects and relatively recent studies have also linked statins to other conditions (Shah and Goldfine, 2012). Patient adherence has also been shown to be an issue (Maningat et al., 2013) since adherence decreases with time and around half of patients discontinue statin therapy within the first year. Prescription of statins is being debated as a public health issue with also, significant implications in the budgets of national health systems.

The QSP model presented in this paper integrates information from the physiology of the patient, hemodynamics, the dynamics of immune cells and lipids, and the dynamic effect of statins (in this particular case, *simvastatin*), as discussed in Pichardo-Almarza and Diaz-Zuccarini (2016). The description and use of this model exemplifies the combination of different modeling paradigms from a pure modeling point of view whilst also providing a concrete case study of its potential usability as an application software in a clinical environment by assessing the effect of pharmacological intervention combined with biological/physiological or behavioral factors in atherosclerosis progression and treatment in specific patients.

The paper is organized as follows: Section Methods presents a formal description of different mathematical paradigms used to model the disease (i.e., atherosclerosis) and the effect of the statin (simvastatin). Section Results: A Case Study presents a case study with the simulations from the model, showing different case scenarios based on drug administration, patient variability, and medication adherence. Finally, Sections Discussion and Model Validation show the discussion and conclusions of this work.

## Methods

### Modeling the atherosclerotic process

**Figure 1:**
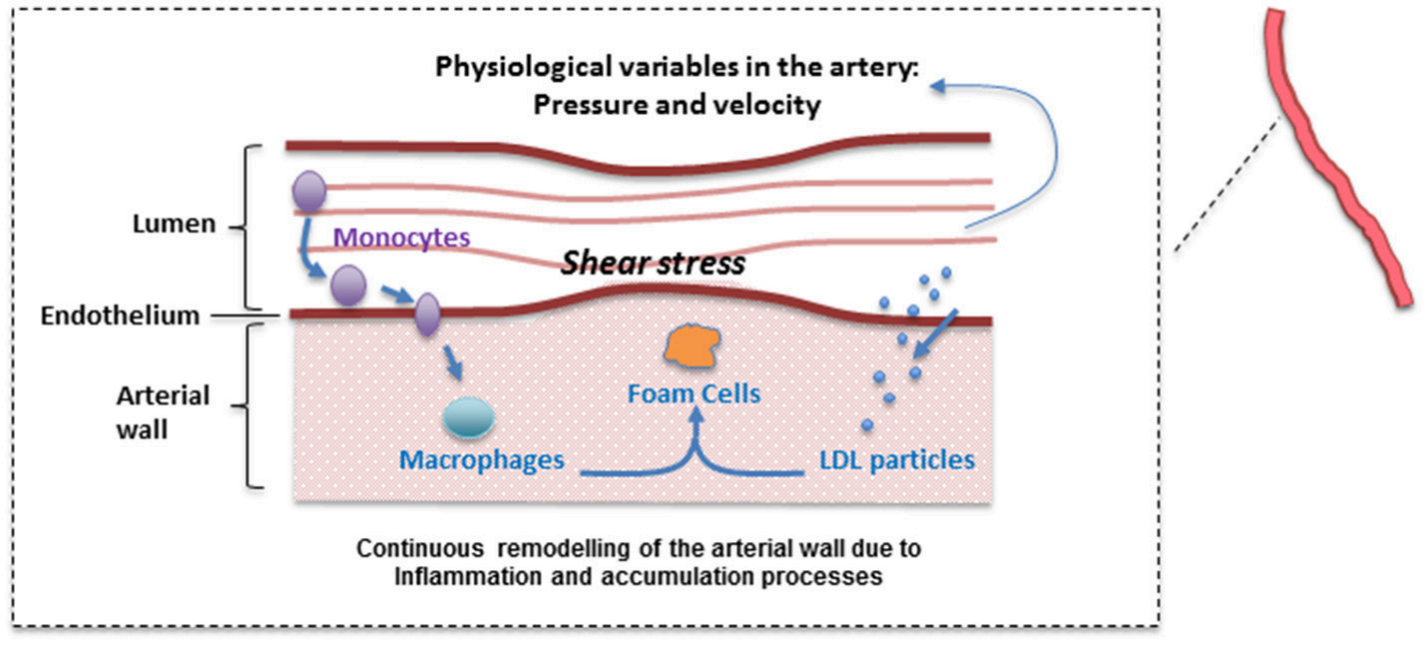
Simplified atherosclerotic process. A plethora of mechanisms at different levels (hemodynamic, cellular, sub-cellular, etc.) are involved in plaque formation. Please note that this is a “positive feedback loop” with nested/coupled processes. Species concentrations in the lumen and the arterial wall (and the WSS) are affected by changes in the arterial wall. Arterial hemodynamics will be affected by arterial remodeling and this in turn, will affect the accumulation of lipids inside the arterial wall.

The main assumption of the atherosclerosis model proposed here is that the penetration of LDL into the intima of the vessel is the main trigger of the disease (Osterud and Bjorklid, 2003), where it is oxidized by free radicals leading to an inflammatory response: monocytes adhere to the endothelium, penetrating into the intima, differentiating then into macrophages (see Figure 1). Due to a chronic inflammatory response, secretion of pro-inflammatory cytokines promotes the recruitment of circulating monocytes, which differentiate to macrophages in the arterial wall. Foam cells are produced due to the uptake of oxidized LDL (ox-LDL) by macrophages, which may be removed by the immune system.

**Figure 2:**
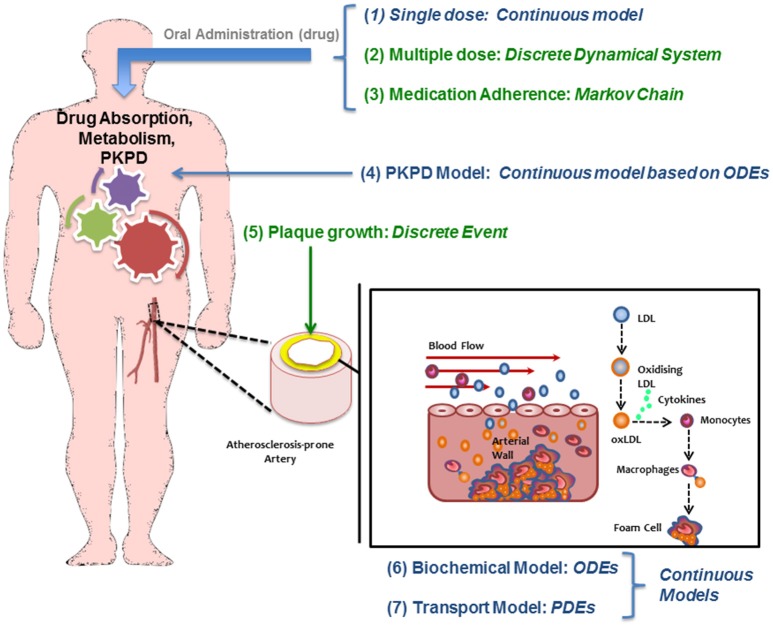
Diagram of the multiscale modeling approach showing how different mathematical approaches are used/needed to study atherosclerosis and its treatment.

A *continuous* modeling approach including the mechanisms described above divides the model in two main parts: a “biochemical” model describing the LDL dynamics (including its oxidation) and the immune response (e.g., pro and anti-inflammatory cytokine effect); and a “mechanical” model describing the plaque growth and the effect of blood flow and mechanical stimuli (wall shear stress) on the endothelium on the evolution of the plaque. In this model, the location of atheroscleorotic plaque is associated with low shear stress; using evidence that suggests the effect of shear stress through endothelial gene expression or by a process of localized inflammation (Cheng et al., 2004). Figure 2 shows a simplified diagram of the processes described by this mathematical model. For each sub-section, the modeling paradigm used (continuous model, Markov model, discrete events, etc.) will be detailed.

#### Hemodynamics and wall shear stress (WSS): a continuous model

The model proposed includes a description of the dynamics of the blood flow. The continuity (1) and Navier-Stokes equations (2) are used for the hemodynamics.

(1) $$\begin{array}{l} \frac{\partial \rho }{\partial t}+\nabla \cdot \left(\rho \;U\right)=0 \end{array}$$

(2) $$\begin{array}{l} \frac{\partial \left(\rho \;\text{U}\right)}{\partial t}=-\nabla p+\nabla \cdot \tau_{\text{i}\text{j}}+\rho g \end{array}$$

Where: *p* = pressure,

∇ · τ_**ij**_ = shear stress tensor,

ρ = density,

*U* = flow velocity.

Transport of molecules from the lumen to the arterial wall can be significantly enhanced or impeded by local flow conditions near the wall and are typically characterized by the WSS.

#### Endothelial behavior and membrane-transport model: a continuous model

The Kedem Ketchalsky's equations (Kedem and Katchalsky, 1958) are proposed to model the LDL flux across the endothelium:

(3) $$\begin{array}{l} J_{v}=L_{p}\left(\Delta p-\sigma \Delta \Pi \right) \end{array}$$

(4) $$\begin{array}{l} J_{s}=P_{0}\Delta c+J_{v}\left(1-\sigma \right)J_{v}\bar{c} \end{array}$$

Where:

*J*_*v*_ = transmural velocity

*J*_*s*_ = LDL flux through the endothelium

*L*_*p*_ = hydraulic conductivity

Δ*p* = pressure difference across the endothelial membrane.

ΔΠ = osmotic pressure difference across the endothelial membrane

*P*_0_ = diffusive endothelial permeability

Δ*c* = solute concentration

$\bar{c}$ = mean endothelial concentration

σ = reflection coefficient.

*L*_*p*_ incorporates the effect of WSS on cellular processes altering transendothelial transport (e.g., intercellular junctions; Chang et al., 2000; Tarbell, 2010). Specific details about how WSS affects the transport of LDL through the endothelium and how the hydraulic conductivity is affected by flow-related variables will not be described here in an effort to be compact; the reader is kindly referred to previous publications (Alimohamadi et al., 2015; Alimohammadi et al., 2016, 2017) for reference.

#### Transport process inside the arterial wall: a continuous model

Macromolecule transport (e.g., LDL) from the lumen to the arterial wall as part of the atherosclerosis progression is typically modeled using a convection-diffusion-reaction (see for example Di Tomaso et al., 2011; Díaz-Zuccarini et al., 2014):

(5) $$\begin{array}{l} \frac{dL_{w}}{dt}=-{\text{u}}_{\text{w}}\cdot \nabla L_{w}+D_{w}L_{w}-r_{w}L_{w} \end{array}$$

Where:

***u*_*w*_** = transmural velocity

*D*_*w*_ = diffusivity of LDL in the wall

*r*_*w*_ = reaction rate constant representing LDL oxidation.

Once LDL is inside the arterial wall it will be oxidized by Reactive Oxygen Species (ROS) triggering an inflammatory response: (1) monocytes will penetrate from circulation and will differentiate to macrophages; (2) macrophages will uptake oxidized LDL to create foam cells that will accumulate as part of the atherosclerotic plaque. All these additional mechanisms related to the LDL oxidation and inflammation are implemented in the integrated multiscale model by using continuous ODEs equations based on mass conservation laws for each biochemical species. This part of the model is also affected by WSS values calculated from the hemodynamic module described above (for more details about the model, see Pichardo-Almarza et al., 2014).

#### Modeling atherosclerotic plaque growth: a discrete event

The atherosclerotic plaque volume (i.e., volume of intima and media layers) is calculated adding the volumes occupied by the different cells and macromolecules (i.e., LDL, monocytes, macrophages, foam cells) inside the arterial wall.

**Figure 3:**
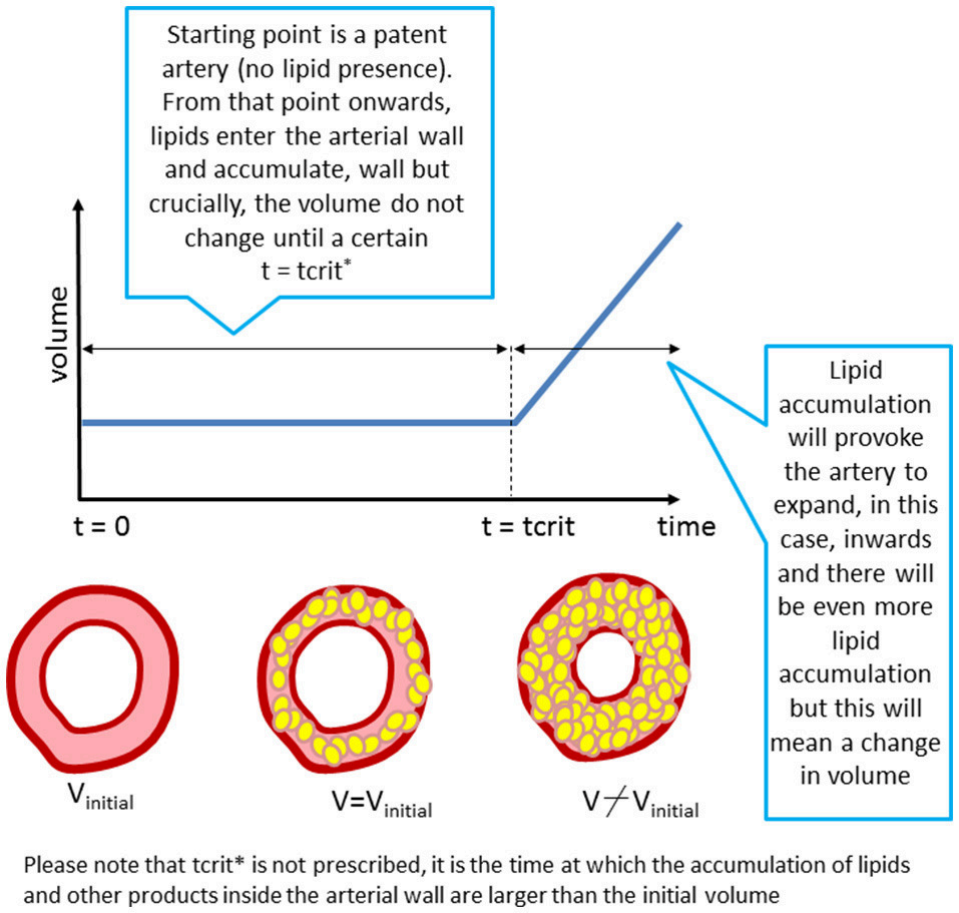
“Growth” as implemented in the model. The arterial lumen (in white) would be remodeled as a consequence of volume growth (inner region of the artery) due to plaque formation and development in time (in yellow).

The formation of the initial stages of the plaque is simulated assuming the lumen will narrow if the volume occupied by all the biochemical species in the wall is bigger than the initial volume of the arterial wall (Figure 3). The amount of foam cells is the key variable to determine this arterial remodeling.

The approach proposed in this work is based on a discrete event in order to pass from “Constant Volume” to “Plaque Growing” in the complete multiscale model.

### Modeling the effect of the drug using a pharmacokinetic/pharmacodynamic (PKPD) model of statins: a continuous model

The continuous PKPD model used in this work is based on a previous published model (Kim et al., 2011). The approach uses a classical compartmental model to describe the PK of Simvastatin and its PD effect on lowering LDL in male volunteers.

**Figure 4:**
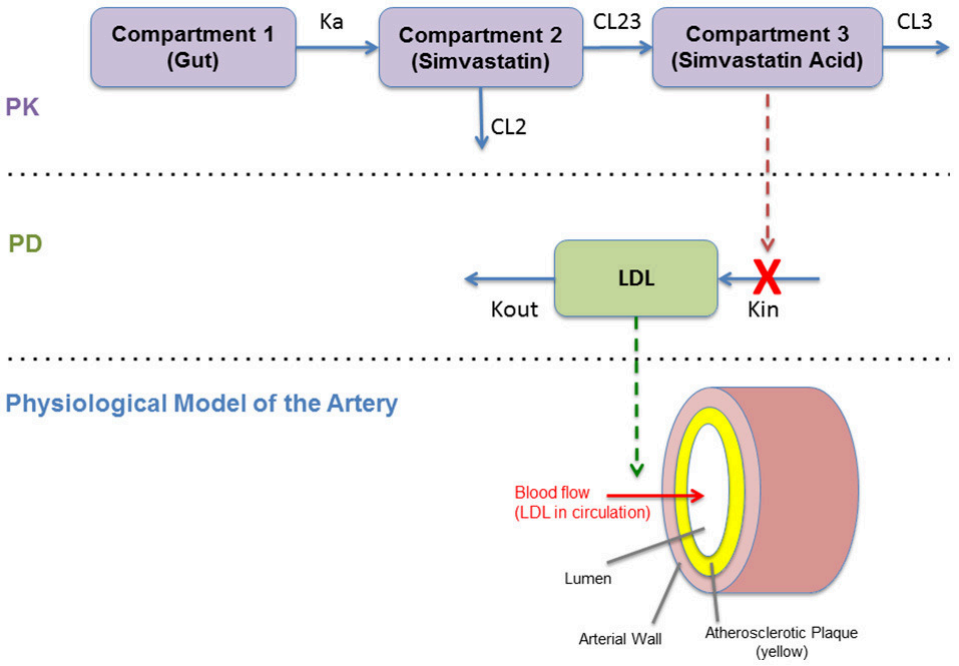
Diagram of the pharmacokinetics/pharmacodynamics (PKPD) model (Kim et al., 2011).

The PK model consists of a parent/metabolite (simvastatin/simvastatin acid) model with first-order absorption and elimination, being a good configuration to fit the PK data. The PD model describes the effect of simvastatin on circulating LDL levels using an inhibitory turnover model with the metabolite (simvastatin acid) as the driver (Figure 4).

The PKPD model is shown in Figure 4. This model reproduces the temporal behavior of the drug (simvastatin) related to its absorption, metabolism, and its pharmacological effect on hepatic cholesterol production and circulating LDL. This continuous model describe these mechanisms mathematically using a set of ordinary differential equations (ODEs) based on a PKPD model with two standard compartments (Kim et al., 2011). This effect on LDL is then translated to the physiological model of the artery that describe the plaque growth based on different mechanisms related to LDL transport (and accumulation) and the inflammatory response (as described in Section Modeling the Atherosclerotic Process).

### Daily dose of statins (multiple dosing): discrete events

**Figure 5:**
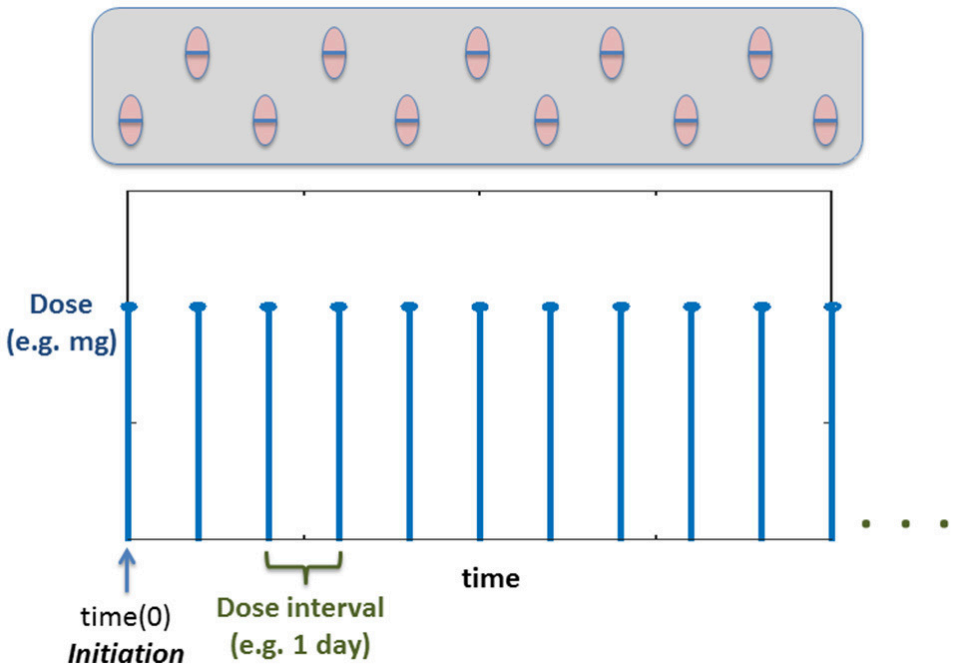
Example time course of how the dose will be administered based on the Discrete Events. Time(0) corresponds to the initiation (Vrijens et al., 2012) (time for the first dose).

Using the previous PKPD model *is not enough* to describe a daily dose of statins. A multiple dose scheme is by nature a discrete model where the amount of drug in the circulation increases at specific times of the day (See Figure 5). Thus, to evaluate the effect of multiple dosing in the current model, a hybrid approach is used, where the dose is modeled as a discrete sequence with a specific amount of drug (modifying the initial conditions of the drug inside the gut compartment in the PKPD model shown in the previous section) and predetermined times. Figure 5 shows an example of the dose sequence mainly *depending on the dose intervals specified by the user*.

### Evaluating medication adherence: a Markov chain model

The previous model of multiple dosing is useful in order to understand the pharmacokinetics of the drugs and their dynamic effect on atherosclerosis. However, the main assumption of the previous model is that the “patient” being modeled is considered to be adherent to his medication following implementation of a given dosing regime, i.e., the patient is taking all his daily doses as prescribed. Lack of patient adherence when treated with statins is a problem widely reported in the literature (Maningat et al., 2013). The computational approach presented here can be used to understand more realistic situations where medication adherence in an issue, in which case, a probabilistic model can be used.

A two state Markov chain is proposed to include the variability on medication adherence in different patients, conditional to the temporal scheme for drug administration proposed in the model, i.e., daily dose of Simvastatin mentioned above.

**Figure 6:**
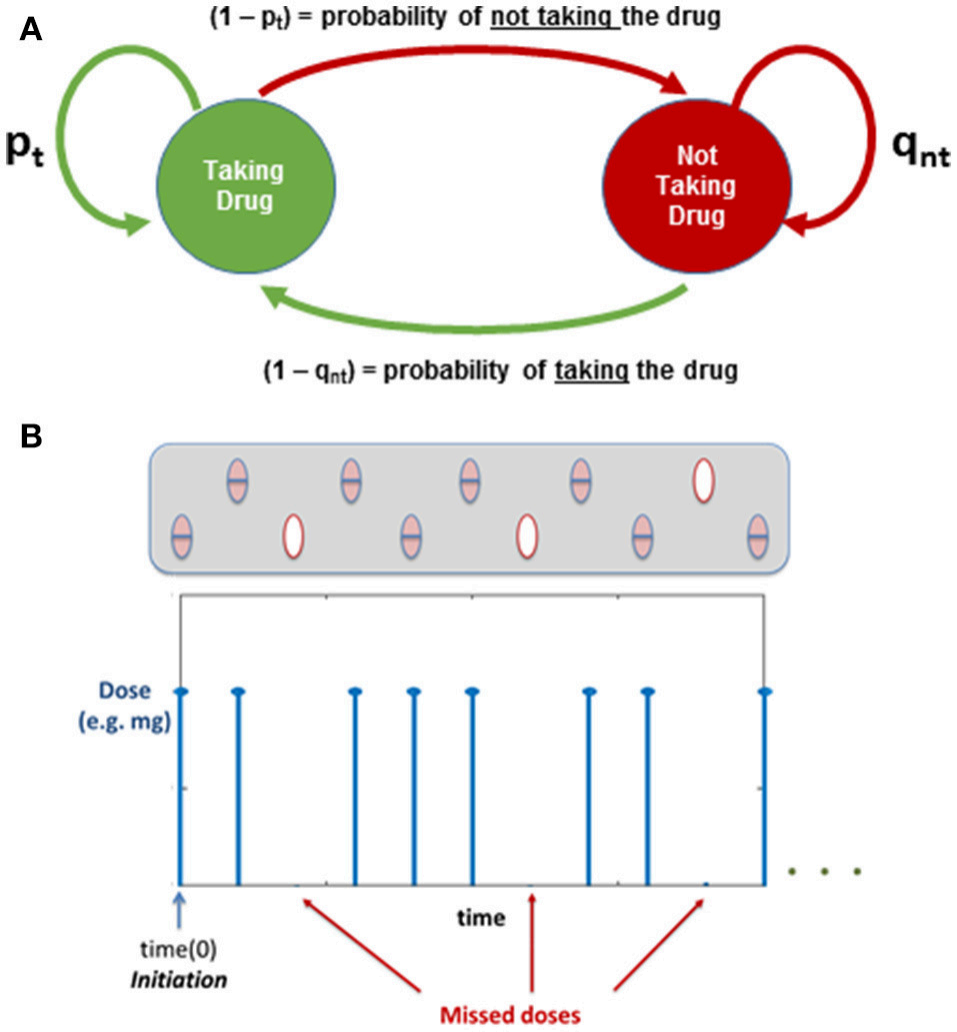
**(A)** Markov Chain: Evaluating Medication Adherence; *p*_*t*_ and *q*_*nt*_ are probabilities based on the previous action (*i-1*). **(B)** Illustration of how the dose regime is affected by the Markov Chain.

Two conditional probabilities of taking or not the daily dose are used to define the two state Markov chain, *based on the patient's behavior the previous time* (see Figure 6).

In this case, probabilities *P*_*t*_ and *Q*_*nt*_ are defined as follows:

(6) $$\begin{array}{l} P(y_{i}=“Taking\;Drug”|y_{i-1}=“Taking\;Drug”)=p_{t} \end{array}$$

(7) $$\begin{array}{l} P(y_{i}=“Not\;Taking\;Drug”|y_{i-1}=“Not\;Taking\;Drug”)=q_{nt} \end{array}$$

From which:

(8) $$\begin{array}{l} P(y_{i}=“Not\;Taking\;Drug”|y_{i-1}=“Taking\;Drug”)=1-p_{t} \end{array}$$

(9) $$\begin{array}{l} P(y_{i}=“Taking\;Drug”|y_{i-1}=“Not\;Taking\;Drug”)=1-q_{nt} \end{array}$$

Which yields to the Markov transition matrix:

(10) $$\begin{array}{l} P=\left[\begin{array}{cc} p_{t} & 1-p_{t}\\ 1-q_{nt} & q_{nt} \end{array}\right] \end{array}$$

Implementing the Markov chain described above allows the user to incorporate into the model the effect of adherence: initiation and implementation (i.e., the patient following the regime or missing some doses), which could be used to understand changes in plaque progression depending on individual behaviors. Figure 6B shows an illustration of how the dose regime can be affected when implementing the Markov Chain to study medication adherence.

### Computational algorithm

**Figure 7:**
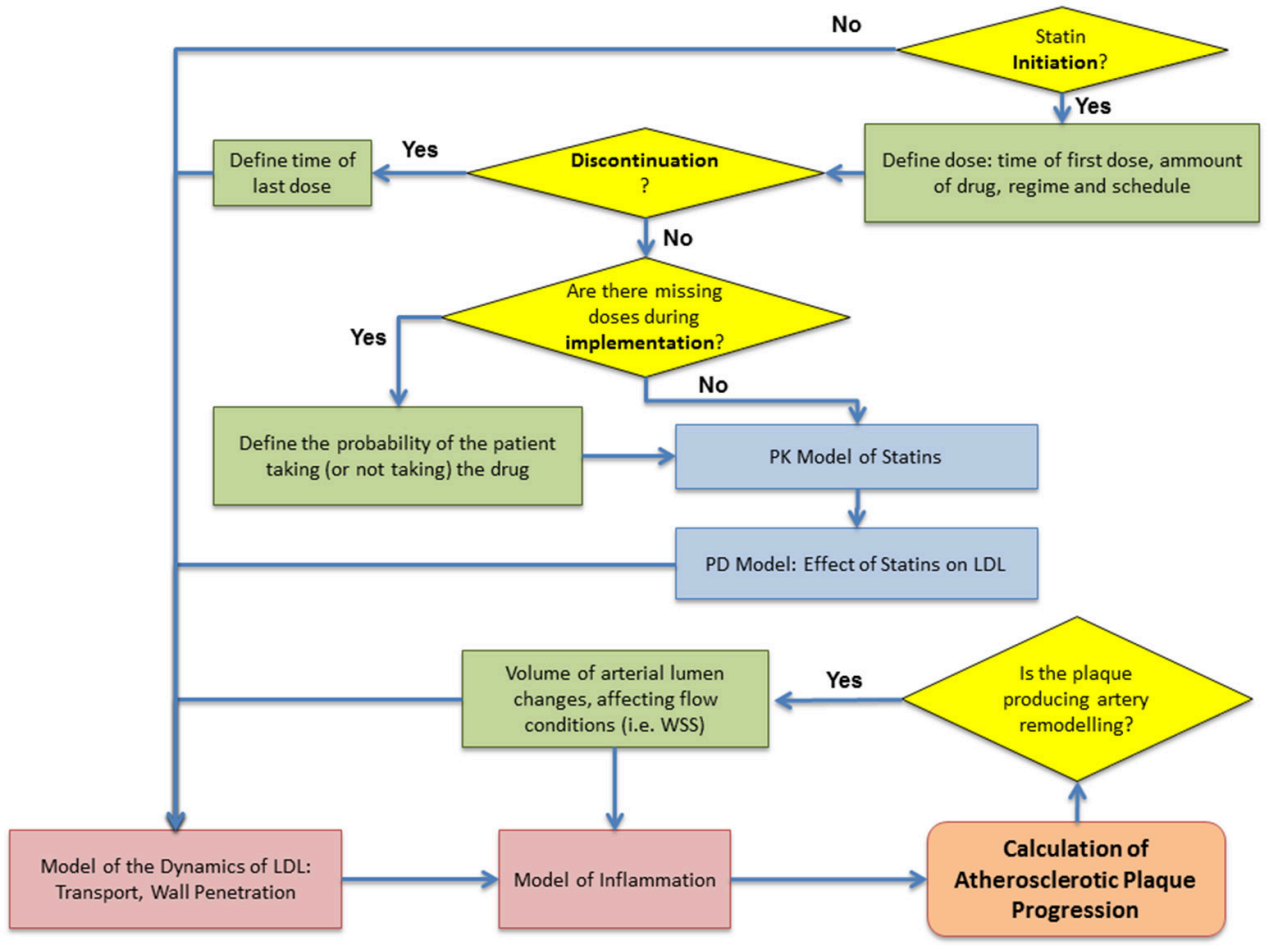
Flowchart of the computational algorithm used to simulate the multiscale model. Patient adherence: *initiation, implementation and discontinuation* defined as proposed by Vrijens et al. (2012).

The integration of the different (sub) models presented previously is summarized in Figure 7. The aim of the complete multiscale modeling framework is allowing the user to have enough flexibility in the implementation in order to simulate and test a variety of different case-scenarios, from simulating patients with no treatment or non-adherent to their medication (exploring only atherosclerotic plaque progression) to group of patients with a given adherence and persistence (e.g., taking all the prescribed drug or missing some of the doses in the whole treatment).

## Results: a case study

**Figure 8:**
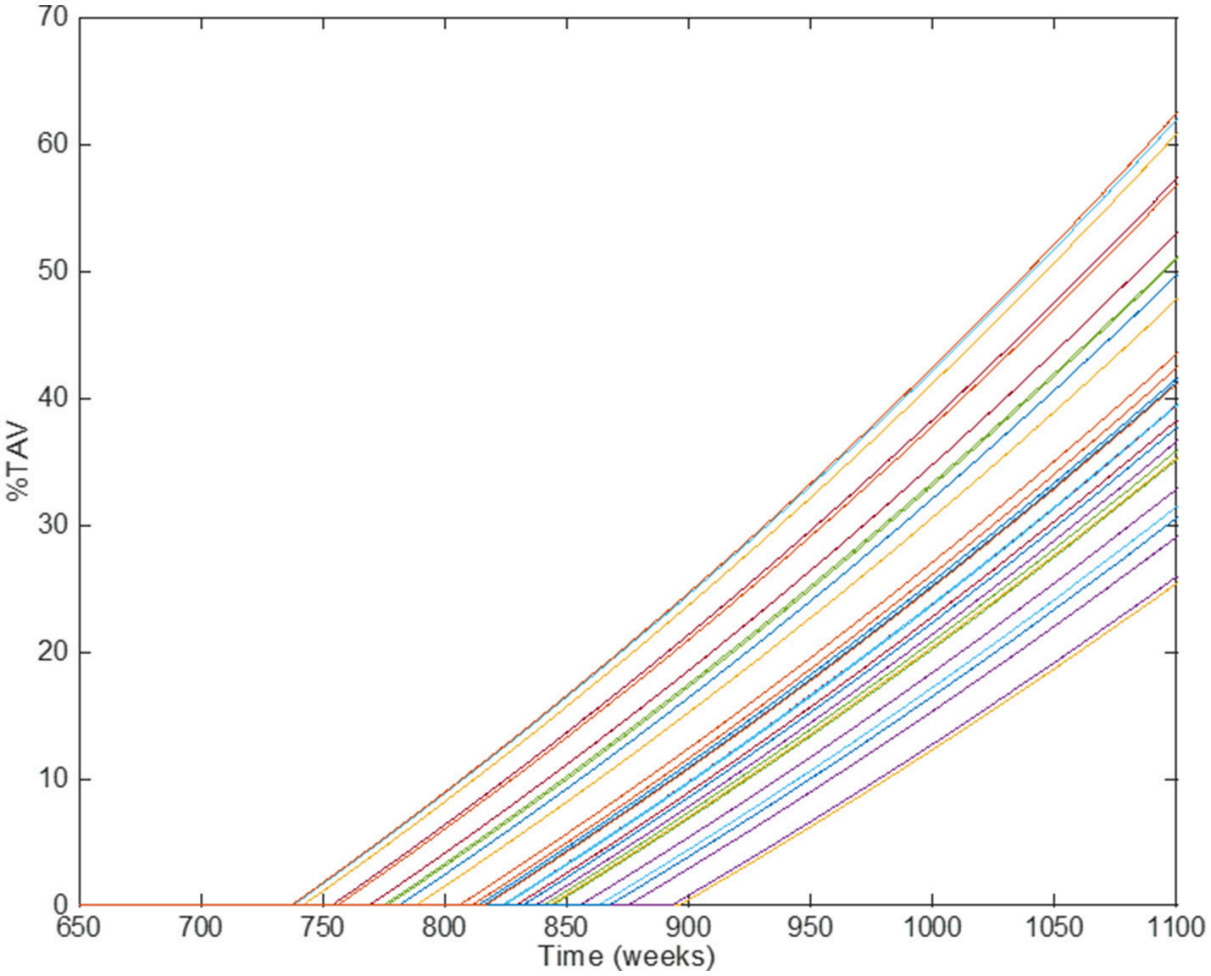
Comparison of simulation results using *different individual parameters* in the original physical–based model of atherosclerosis. Different lines show the results of individual patients' trajectories (plaque growth for individual patients); the time when the plaque starts to growth (time for transition) can be different for different individuals.

The multiscale model proposed here is the result of integrating several approaches to capture and understand the progression of atherosclerosis and how statins helps to inhibit plaque development. The clinical endpoint is related to how the plaque size changes dynamically. The fact that vascular remodeling under these hypotheses is akin to a “positive feedback loop” with nested/coupled processes, means that the dynamic behavior of the plaque has an effect on all the components of this model, mainly because changes in the arterial wall affect WSS and also the concentration of biochemical species in the artery and the lumen (see Figures 1, 2). The complete model is composed of four main components which were coupled to describe all the relevant mechanisms and interactions: (1) Mechanical properties of the artery, including blood flow; (2) Macromolecule and immune cell transport through the endothelium; (3) PK model of statins (e.g., considering administration and metabolism of the drug) and their pharmacological effect; (4) simulation and analysis of adherence. Parameter values in the different parts of the model can be modified in order to study the variability of plaque growth in a given patient population. Table 1 shows a summary of the physiological parameters used in this model; more details can be found in Pichardo-Almarza et al. (2014). Figure 8 shows how changes in some model parameters (please see below) produce different dynamics for plaque growth for a patient group. In particular, it is possible to identify the individual time when the plaque starts to grow.

**Table 1:** Quantitative systems pharmacology model: parameter values for a *Typical Patient*.

**Symbol**	**Quantity**	**Value**
**MODEL OF ATHEROSCLEROSIS**		
*R_*lumen*_* (mm)	Initial lumen radius	3.0
*k_*m*_* (m^3^ cells^−1^ s^−1^)	Foam cell formation constant	9.25 × 10^−24^
*d_*m*_* (s^−1^)	Diffusion of monocytes out of the plaque	5.75 × 10^−6^
*d_*lox*_* (s^−1^)	Diffusion of LDL out of the plaque	2.4 × 10^−5^
*d_*M*_* (s^−1^)	Diffusion of macrophages out of the plaque	5.75 × 10^−6^
*d_*F*_* (s^−1^)	Diffusion of foam cells out of the plaque	5.75 × 10^−6^
*r_*w*_* (s^−1^)	LDL oxidation rate constant	3 × 10^−4^
μ (Pa s)	Blood viscosity	0.004
ρ_1_ (s^−1^)	Rate of differentiation of monocytes	1.15 × 10^−6^
Δ*p* (Pa)	Endothelial pressure difference	2,400
*Q*(l/s)	Blood flow	0.0075
*m_*lumen*_* (cells/l)	Concentration of monocytes in blood	5.5 × 10^8^
σ	Endothelial reflection coefficient	0.997
**PKPD MODEL**		
CL2(L/hr)	Clearance compartment 2	1,740
V2(L)	Volume compartment 2	8,980
CL3(L/hr)	Clearance compartment 3	383
V3 (L)	Volume compartment 3	1,190
Ka (1/hr)	Absorption rate constant	2.76
K_in_ (nmol/L/hr)	Production rate of LDL	29.52
Emax	Max. effect due to the drug	0.489
EC_50_ (ng/mL)	Blood concentration at half max. effect	0.0868
LDL_baseline_ (nmol/L)	Baseline LDL	1,400

**Figure 9:**
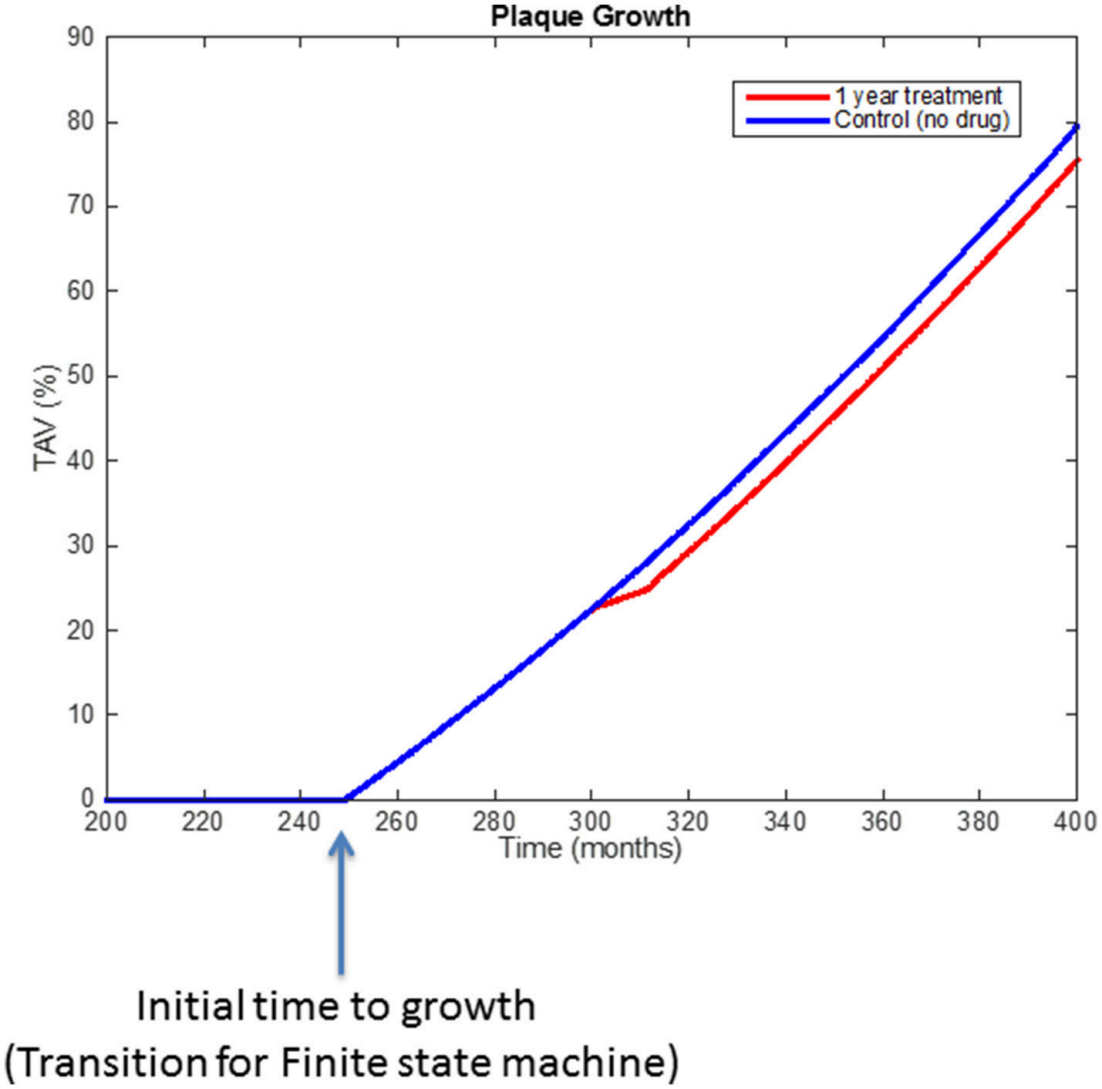
Comparison of simulation results using the original physical-based model of atherosclerosis (blue line) and the effect on plaque growth when a one-year statin treatment is considered on the model (red line).

Figure 9 shows the behavior of the model (i.e., rate of plaque growth, measured in terms of volume) for an individual patient without any treatment (control, blue line) vs. the effect of the same patient on the drug (dotted red line). Changes in the growing slope were previously compared with the plaque growing trends obtained from Jensen et al.'s study (Jensen et al., 2004) and already presented in Pichardo-Almarza et al. (2014). Decreasing circulating LDL due to the simvastatin therapy has an additional effect on decreasing the transport of LDL through the arterial wall, which also affects the production of foam cells, leading to a slower plaque growth rate. Please note that the %TAV is the change (%) of Total Atheroma Volume, calculated by the model.

Furthermore, the model is helpful to explore how blood flow has an effect on the initiation and progression of the atherosclerotic plaque. The pattern of blood flow (and specific properties as WSS Tricot et al., 2000) will be affected due to changes in lumen diameter owing to plaque growth. Also endothelial cells will respond to signals induced by changes in WSS (Dimmeler et al., 1999; Tricot et al., 2000). Thus, the shear stress regions of the atherosclerotic plaque will remain atheroprone.

**Figure 10:**
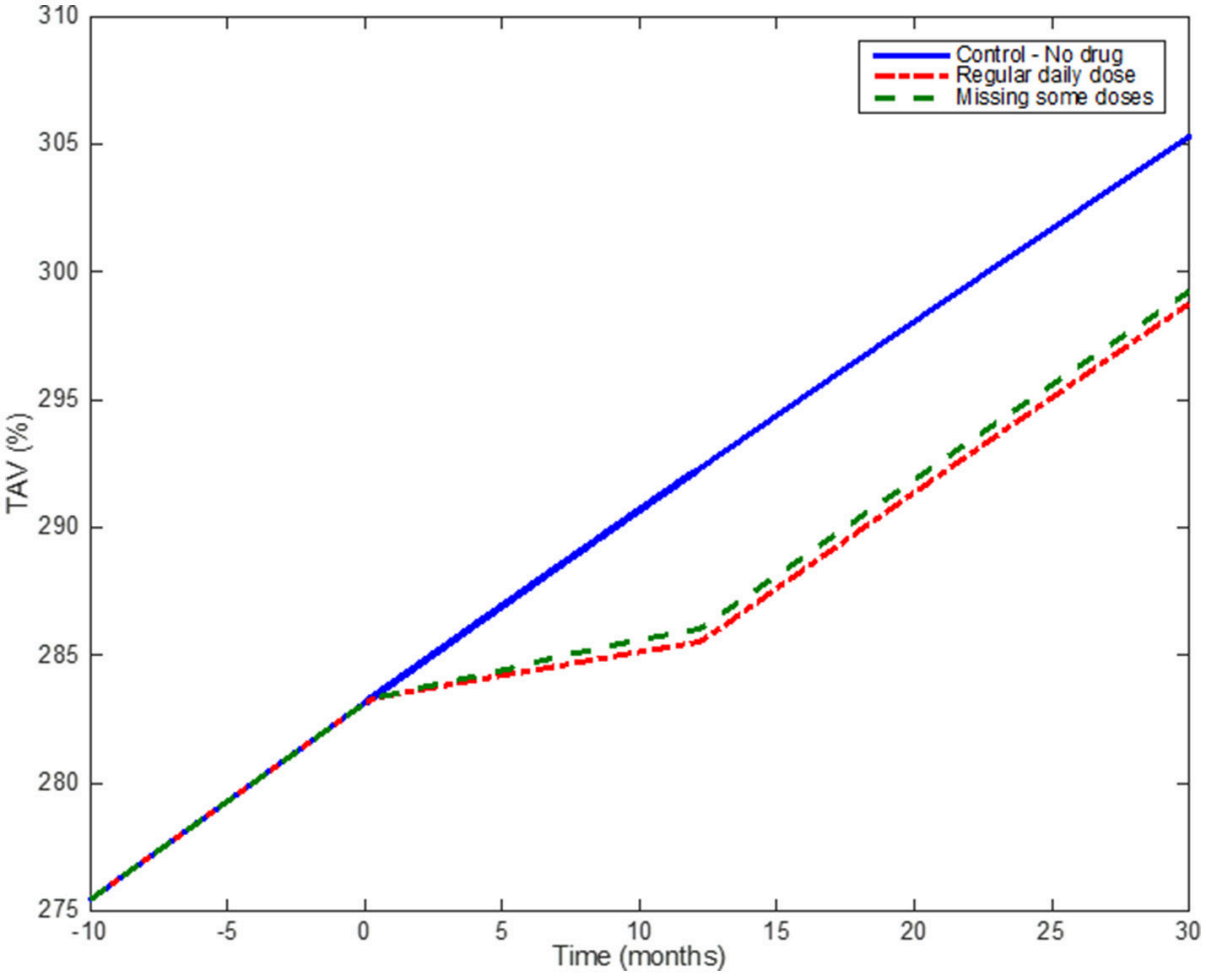
Medication adherence: Simulation results comparing the effect of taking statins on a regular basis and missing some doses according to the output of the Markov Chain. The blue (solid) line shows the model when no drug is administered; the red dotted line shows the effect of one-year treatment with Statins (considering the patient taking the drug regularly); the green line shows the effect of a patient under the same Statin treatment but missing ~10% of the total number of doses.

An example of the effect of medication adherence on the progression of the atherosclerotic plaque is shown in Figure 10, which demonstrates how, by using a discrete probabilistic approach (Markov Chain) in the model, the user can compare different scenarios between a control group, a regular group (“adherents”) and a “less adherent” group (missing some doses during implementation). In the example, adherent patients are considered as patients taking all the doses during a year (so the Markov Chain model for dosing is not required); the “less adherent” patients correspond to a case where the Markov Chain model (Figure 6) was used with *p*_*t*_ = 90% and *q*_*nt*_ = 25%. These different scenarios allow evaluating the influence of missing some daily doses on the overall effect of a treatment prescribed for a year. Statins seems to still have a similar effect on plaque progression when the patient misses some of the doses, however it is clear that the effect on reducing the plaque growth is less than in the regular case.

## Discussion

Results of the coupling between the different mathematical approaches to describe the pharmacokinetics/pharmacodynamics of statins and different atherogenic mechanisms in the artery show how changes in different parameters of the model, able to represent individual patient trajectories, can be used to evaluate different scenarios to study atherosclerotic plaque growth. Comparing the results with the clinical endpoints reported by Jensen et al. (2004) shows how this kind of systems models can be calibrated with clinical data reproducing the dynamic response of plaque growth for untreated patients and patients treated with simvastatin (Pichardo-Almarza et al., 2014).

The main aim of this work is to use an adaptable approach and different formalisms to develop a model that provides enough flexibility to the user in order to create different case scenarios to test physiological and pharmacological hypotheses in the study of atherosclerosis, the effect of statins on the progression of the disease and the effect of patient adherence (with multiple possible scenarios) to treatment for different patient trajectories. One of the main advantages of this approach is the possibility to expand or simplify the model according to different assumptions or requirements, made by the user. The approach is modular (i.e., the model can be decomposed in different parts), which means the user has the possibility to modify (or improve) a given module related to a specific physiological/biological process in order to improve simulation results for further analyses and/or validation. Just to provide an example, even if the analyses reported in this paper only show the effects of simvastatin, *other cholesterol-lowering drugs could also be considered* using the same modeling approach, just by changing the pharmacological properties of the drug. In this case, the PK model could be substituted by previous models published in literature, more specifically any of the models proposed by Faltaos et al. (2006) or Vargo et al. (2014) could be “plugged in” the multiscale computational framework in order to study the effect of atorvastatin. These features make this work suitable for software development.

Atherosclerosis and the effect of statins in humans have been analyzed in the past using conventional pharmacokinetics and pharmacodynamics (PKPD) modeling approaches (Kim et al., 2011; Wright et al., 2011). In particular, Wright et al. (2011) explored the influence of variable compliance, circadian cholesterol production and simvastatin dosing time on the reduction of low-density lipoprotein (LDL). This work mainly showed the impact of medication adherence on the effect of statins on decreasing LDL levels in blood, however, to our knowledge there are no previous attempts where a multiscale approach as the one proposed here has been used to include models of downstream biology/physiology in order to study the effect of these drugs on the disease development and progression (i.e., atherosclerosis).

Our work shows that these models can be combined with a multi-scale approach, expanding the biological/physiological mechanisms and providing a better description of specific pathways related to the disease, the PKPD properties of the drugs and patient behavior with respect to medication adherence. A quantitative systems pharmacology (QSP) approach was applied, which combines PKPD modeling and physiological/disease modeling based on fluid dynamics and mass transport, providing a holistic computational approach to describe the dynamic interaction between different elements in the artery, the plaque, and the drug.

## Model validation

**Figure 11:**
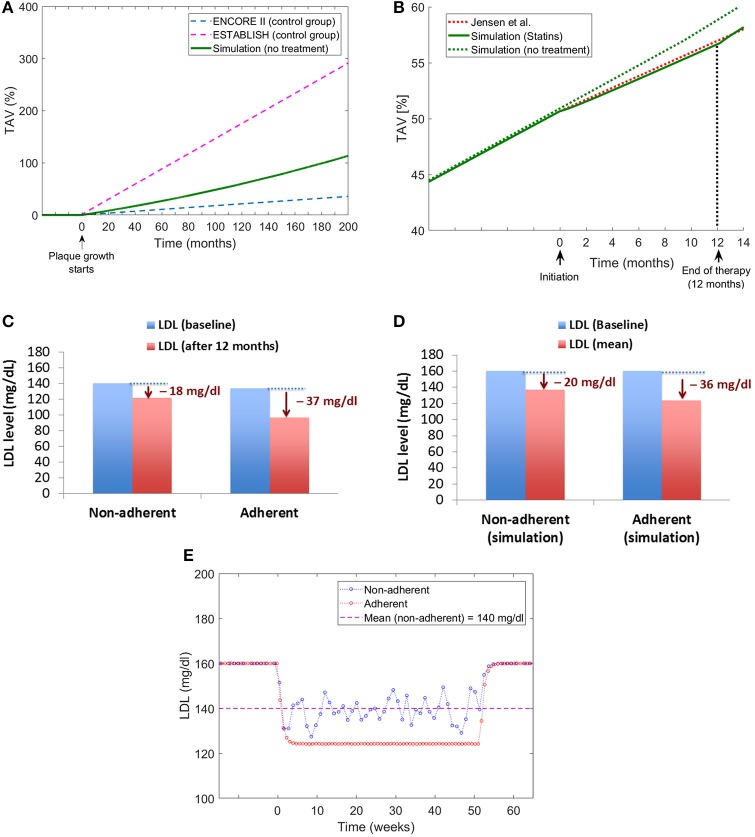
Model validation. **(A)** Comparison of plaque growth simulated by the QSP model with trends reported by clinical trials [ENCORE II (Lüscher et al., 2009) and ESTABLISH (Okazaki et al., 2004)]. **(B)** Comparison of the plaque growth simulated by the model with the change in plaque growth trend reported by Jensen et al. (2004). **(C)** Changes in LDL levels for adherent and non-adherent patients after 12-month statin treatment reported by Kazerooni et al. (2013). **(D)** Changes in LDL levels obtained from the QSP model for adherent and non-adherent patients. **(E**) Time course of LDL changes due to treatment for adherent and non-adherent patients obtained from the QSP model simulations.

The multiscale QSP model proposed was validated using available data related to changes in Total Atheroma Volume (%TAV) for control groups and patients treated with statins. When modeling patients without treatment, the model estimates a plaque growth in the middle of the growing slopes reported for the control groups in the ENCORE II (Lüscher et al., 2009) and ESTABLISH (Okazaki et al., 2004) clinical trials (see Figure 11A). When implementing a statin treatment in the QSP model, the change in the plaque growth slope due to the pharmacological effect of the drug match very well the changes reported by Jensen et al. (2004; see Figure 11B). To the best of the author's knowledge, there are no additional clinical studies in the literature that can be used to compare the effects of statin adherence on atherosclerotic plaque progression, however, there is evidence that missing doses during statin implementation has an impact on LDL levels (Kazerooni et al., 2013; Chen et al., 2015), for example in the study from Kazerooni et al. (2013) it was reported that in a veteran population of more than five thousand patients, there was on average a decrease of LDL levels of 37 mg/dl for adherents and 18 mg/dl for non-adherents after 12 months of treatment (see Figure 11C); the QSP model reproduces similar differences in LDL for these two groups of patients when comparing individuals with total adherence and a group of patients with a low proportion of taken doses with respect to the original prescription (i.e., around 50% of the prescription; see Figure 11D). An additional advantage of the QSP model is the possibility of seeing the variations (i.e., oscillations) in the LDL time course due to missed doses (see Figure 11E), something that is not reported in clinical or epidemiological studies. This clearly shows that it will be more useful for future model development if the data coming from the clinic also includes information about the implementation of the drug for each patient (e.g., missing doses, etc.), a variable that is not normally recorded in clinical studies of statins.

## Limitations

It is important to highlight that the lack of available (clinical) data for adherence to compare against constitutes a limitation that needs to be acknowledged. The authors hope that as this type of approaches grow, relevant and rich datasets can be collected to underpin the crucial developments of this type of technology. Regrettably, this is not the case right now but we are hopeful that in the future, longitudinal and multiscale datasets will be collected for adherence purposes. In fact, it can be argued that novel approaches such as the one presented here where the disease in question (and not a surrogate biomarker) is the endpoint, can pave the way for future studies by making a strong case for data collection in longitudinal and coherent datasets at multiple scales.

Another limitation regards the atherosclerosis model presented here. This model is obviously simplified; atherosclerosis is a complex and multifactorial disease with contributors at different levels of biological organization. In particular, the genetic component was not included, which might affect the results for one specific patient trajectory (an individual patient). Additionally, the model does not currently consider the pleiotropic effects of statins (but could consider it in the case of an expanded model). All this been said, the results at the population level should still be representative and in fact, there is good agreement between the simulation results and the published data of epidemiological studies on statins as described above in the “Results” and “Model Validation” sections.

There is also a simplification in the hemodynamics of the system, notably through the use of a Newtonian model for blood, Poiseuille flow and “idealized” arterial conduits. The model only considers drug effect on the early stages of atherosclerosis (LDL, monocyte infiltration, foam cell formation, etc.) but additional mechanisms could be included, depending on data availability.

Finally, more research is necessary to understand the consequences in terms of patient adherence of side-effects, lifestyle and the environment, especially for long term treatment. This is clearly out of the scope of this paper but we recognize that this might have a strong impact in the adherence regime for individual patients. If this could be accounted for in a more mechanistic fashion, it would provide a better understanding of the weighing of different aspects (besides biology) that affect whether or not a patient is likely to continue with their treatment as prescribed and the effect this might have at the clinical endpoint.

Despite these limitations, the work presented here is strongly anchored in the state of the art and current knowledge in this area and we believe it provides a tool to help understand the overall effect of patient-adherence in the case of statins.

## Conclusions

Data from controlled clinical trials and observational studies have been reporting low adherence to statin medication (Rash et al., 2016). These studies have shown a continuous decline of adherence to statin therapy after the first prescription with ~50% patient treated stopping the use within the first year of treatment (Lemstra et al., 2012). Developing computational tools allowing to estimate the benefits associated with statin therapies in combination with patient behavior might help to understand the effect of adherence in clinical endpoints and the possibility to test case scenarios for specific patient populations

The approach proposed in this work for modeling and simulation in physiology and pharmacology requires the integration of different mathematical techniques and formalisms in order to capture the hybrid nature of the problems and mechanisms related to cholesterol-lowering drugs and cardiovascular diseases.

Continuous models based on differential equations are particularly useful to describe the physics of chronic diseases such as atherosclerosis. However, additional, discrete events modelsare needed to provide a description of specific physiological mechanisms, e.g., plaque growth.

Here, a hybrid approach was used to understand the evolution of atherosclerotic plaque in individualized simulations, combining different mechanisms, and hypotheses related to the effect of specific dosage regimes (e.g., daily dose) on patients treated with statins. The detailed description of diverse formalisms used helps to explain and understand different scenarios where the interplay between medication adherence and pharmacological intervention could have an influence on treatment efficacy and disease progression.

The work proposed in this paper is modular and adaptable in essence. One of the highlights of this approach is that it allows *targeted modifications* (extensions or simplifications) of specific parts of the model without affecting the implementation of the whole, which is an interesting feature for software development. This attribute was particularly useful to build “virtual” populations in order to evaluate the predictive effect of the model when compared with clinical trials (Pichardo-Almarza et al., 2014). The proposed simulation framework allows the possibility to design more complex simulation strategies (e.g., Monte Carlo Simulations) with specific tasks in mind, in order to test specific biological/physiological/pharmacological hypotheses in the study of atherosclerosis. Finally, this work can be of interest to the biological, pharmacological and the medical community as it allows to test hypotheses and “what-if” scenarios related to drug administration, patient adherence and atherosclerotic development in a comprehensive way. Although, reporting on software testing is not within the scope of this paper, the framework presented here is currently being evaluated by clinical pharmacology groups in UCL and it will be the object of future work.

## Author contributions

CP designed the study, coordinated the study, carried out the data analysis, mathematical modeling and simulations and drafted the manuscript; VD helped to design the study, coordinated the study and drafted the manuscript. All authors gave final approval for publication.

### Conflict of interest statement

The authors declare that the research was conducted in the absence of any commercial or financial relationships that could be construed as a potential conflict of interest.
